# Factors Associated with Patients’ Ratings of Hospitals among Japanese Inpatients: A Cross-sectional Study

**DOI:** 10.31662/jmaj.2022-0176

**Published:** 2023-04-13

**Authors:** Haruna Nishio, Sachiko Ohde, Noyuri Yamaji, Osamu Takahashi

**Affiliations:** 1 Graduate School of Public Health, St. Luke’s International University, Tokyo, Japan; 2 Center for Clinical Epidemiology and Health Technology Assessment, St. Luke’s International University, Tokyo, Japan

**Keywords:** HCAHPS, Hospital Consumer Assessment of Healthcare Providers and Systems, patient satisfaction, patient rating, hospital care quality, quality indicator, patient-reported outcome measures, cross-sectional study

## Abstract

**Introduction:**

The evaluation of hospital performance often receives great attention. Hospitals refer to patient ratings to undertake quality-improvement activities. However, little is known about the factors that contribute the most to these patient ratings. This study aimed to investigate the association of relevant factors, such as doctors’ and nurses’ performance, with patients’ ratings of hospitals, using the Hospital Consumer Assessment of Healthcare Providers and Systems (HCAHPS^Ⓡ^) questionnaire.

**Methods:**

A cross-sectional study was conducted among patients who were hospitalized in Japan, from January 2020 to September 2021. Patients’ hospital rating scale scores between 0 and 10 were collected and dichotomized. A score of 8 or higher was defined as a high rating. A multivariate logistic regression analysis was conducted to investigate the association between patients’ ratings of the hospital and other items in the HCAHPS^Ⓡ^ questionnaire.

**Results:**

The frequency of patients’ high and poor hospital ratings were 207 (69%) and 93 (31%), respectively, of 300 respondents. A significant association was observed for the patient’s age (adjusted odds ratio (AOR): 1.02; 95% confidence interval (CI): 1.00-1.04), doctor’s communication (AOR: 10.47; 95% CI: 3.17-34.58), and discharge planning (AOR: 3.53; 95% CI: 1.96-6.36) with a positive patient rating of the hospital.

**Conclusions:**

An emphasis on doctor communication and discharge planning is essential in improving patients’ ratings of hospitals. Further research is needed to determine the factors that contribute the most to patients’ ratings of hospitals.

## Introduction

Achieving high levels of healthcare has been of great importance for hospitals worldwide. The evaluation of hospital performance has been an essential factor for improvements in medical services and quality of care. At the World Health Assembly held in May 2017, the World Health Organization advocated people-centered care (PCC), an approach to care that consciously incorporates the perspectives of individuals, families, and communities, viewing them as participants and beneficiaries of a reliable health system that addresses their needs and preferences in a humane and holistic manner ^(1), (2)^. In recent years, awareness of patient-, individual-, and people-centered healthcare policies has been growing globally, and efforts to evaluate the quality of healthcare systems and providers from the patient’s experience and perspective are growing in popularity ^(3), (4), (5), (6)^. A patient’s experience has been considered one of the central pillars of quality in healthcare and is used worldwide as a tool to assess the quality of healthcare professionals ^(7)^. In addition, a systematic review has found that patient experience is positively associated with clinical effectiveness and patient safety ^(8)^. Hospitals use patients’ ratings of the hospital and healthcare that they have received, to undertake various quality-improvement activities. Although attempts have been made to determine the aspect of healthcare that contribute most to patient outcomes, the factors that contribute most to the patient ratings are still unknown. For instance, many studies have demonstrated that doctor-patient communication and nurse-patient communication are important factors in improving patient satisfaction ^(9), (10)^, with a study suggesting that poor communication skills by healthcare providers lead to dissatisfaction of the patients’ overall care at the hospital ^(10)^. Another study concluded that nonverbal communication and perceived empathy and competency were more significant factors that affected patient satisfaction ^(11)^, indicating that both verbal and nonverbal communication skills are essential in improving patient satisfaction. Enhancing communication also improves patient safety ^(12)^. However, in other cases, for example, communication with families of patients with chronic critical illnesses, satisfaction of care, in the context of anxiety or depression, did not improve with healthcare providers’ communication, but instead increased the risk of posttraumatic stress disorder ^(13)^. Another factor contributing to patient satisfaction is discharge communication. Early involvement in discharge planning benefits patient satisfaction ^(14)^, and providing structured discharge planning is effective in improving the patients’ health status, self-efficacy, and satisfaction ^(15)^. A good hospital environment, for example, having more staff per bed ^(16)^ and the cleanliness of the hospital ^(17)^, also influenced patient satisfaction. From previous research, we have found many attributes to patient satisfaction, such as efficient communication by hospital staff, discharge planning, and a good hospital environment, but results were controversial. Thus, an investigation of such factors will lead to a deeper understanding of and improvement in PCC ^(1)^. As the core of healthcare quality has relied heavily on PCC in recent years, a better understanding of patients’ experiences at the hospital and an understanding of the factors that influence their experiences are of great importance.

Therefore, we have sought to determine the factors that are most likely to contribute to patients’ ratings of hospitals, by utilizing the Hospital Consumer Assessment of Healthcare Providers and Systems (HCAHPS^Ⓡ^). We chose the HCAHPS^Ⓡ^ instrument as this questionnaire comprehensively covers a wide range of factors, including the communication of nurses and doctors, hospital environment, and patients’ experience in the hospital at different time frames, for example, during their stay and at discharge. The comprehensiveness of this questionnaire makes it ideal to assess the factors that most contribute to patients’ ratings of hospitals in an exhaustive manner. Moreover, as the HCAHPS has been translated and used in various languages worldwide, it is expected that validation of the Japanese translation of the HCAHPS questionnaire will enable its use to survey patient satisfaction in Japan and provide material to recommend and further increase patient-centered healthcare policies.

The objective of this study was to determine the factors that most likely contribute to patients’ ratings of hospitals using the HCAHPS questionnaire while examining the degree of each factor, such as doctors’ and nurses’ communication and patient discharge planning, in relation to patient outcomes in an exploratory manner.

## Materials and Methods

### Hospital consumer assessment of healthcare providers and systems (HCAHPS^Ⓡ^)

We chose to use the HCAHPS^Ⓡ^ questionnaire to conduct this study. The HCAHPS was initially developed in the United States of America (USA) in 2006 by the Agency for Healthcare Research and Quality (AHRQ) in response to a request by the Centers for Medicare and Medicaid Services (CMS) ^(18)^. The HCAHPS^Ⓡ^ questionnaire consists of three rating scales, the “HCAHPS Composites,” “Individual Items,” and “HCAHPS Global Items,” and seven items related to individual characteristics (Q23-Q29). The questionnaire contains 19 core questions on critical aspects of patients’ hospital experiences, such as communication with nurses/doctors and questions on the hospital environment. The HCAHPS Composites comprise six items: (1) “Communication with Nurses” (Q1-Q3); (2) “Communication with Doctors” (Q5-Q7); (3) “Responsiveness of Hospital Staff” (Q4, Q11); (4) “Communication about Medicines” (Q13, Q14); (5) “Discharge Information” (Q16, Q17); and (6) “Care Transition” (Q20-Q22). The HCAHPS Individual Items include (1) “Cleanliness of Hospital Environment” (Q8) and (2) “Quietness of Hospital Environment” (Q9). The HCAHPS Global Items include (1) “Hospital Rating” (Q18) and (2) “Recommend the Hospital” (Q19). The Global Items include (1) “Overall Rating of Hospital” (Q21) and (2) “Willingness to Recommend Hospital” (Q22). Previous studies suggested that the HCAHPS^Ⓡ^ questionnaire can be used to assess patients’ care experiences in various countries ^(19), (20), (21)^. The original HCAHPS^Ⓡ^ has been officially translated into Spanish, Chinese, Russian, Vietnamese, Portuguese, and German ^(22)^, as well as published as papers translated into Arabic ^(20), (23)^, Tagalog ^(24)^, and Malay ^(21)^. All translations were conducted by following established guidelines ^(25)^.

Before starting this study, we obtained permission and received documents for the translation of the HCAHPS^Ⓡ^ from the American AHRQ’s Consumer Assessment of Healthcare Providers and Systems (CAHPS^Ⓡ^) program. We strictly followed the standardized translation procedure developed by the CAHPS^Ⓡ^ Cultural Comparability Team ^(25)^. We launched an expert panel of eight people, including clinicians and faculty in the fields of epidemiology and medicine, to review the translated HCAHPS^Ⓡ^. The entire expert panel finally confirmed the accuracy of the translation by identifying the differences between the two versions. During the translation process, we removed Q27 (Are you of Spanish, Hispanic, or Latino origin or descent?), Q28 (What is your race? Please choose one or more.), and Q29 (What language do you mainly speak at home?) as these questions were related to the cultural context of the USA and did not fit the context of Japan. Additionally, given the differences in education systems between Japan and the USA, we revised the answer frame of Q29 (What is the highest grade or level of school that you have completed?) to reflect the Japanese educational system.

Content validation and face validation of the translated HCAHPS questionnaire were assessed following the guidelines for the cross-cultural adaptation process provided by Squires et al. ^(19)^ to avoid methodological errors ^(26), (27)^. Content validity refers to the extent to which the items on a test represent all aspects of the measured construct. We asked 12 discharged patients from St. Luke’s International Hospital, located in Tokyo, Japan, and eight expert panel members to evaluate the content validity of the translated HCAHPS^Ⓡ^^(19), (28)^, and calculated the average of all the raters’ evaluations at the item-level content validity index (I-CVI) and scale-level CVI (S-CVI) ^(29)^. Face validity refers to the extent to which a test appears to measure what it is intended to measure. We obtained data from the same 12 patients to evaluate the item-level face validity index (I-FVI) and scale-level FVI (S-FVI) ^(29)^. As a result, the S-CVI was 0.99, and the I-CVIs of the individual items ranged from 0.95 to 1.0. The validity of each item and the entire survey was excellent, with the I-CVI being 0.78 or higher and the S-CVI being 0.9 or higher ^(30)^. The S-FVI for clarity was 0.96, and the I-FVI ranged from 0.75 to 1.0. The S-FVI for comprehension was 0.98, and the I-FVI ranged from 0.75 to 1.0. The I-FVI was 0.78 or higher and the S-FVI was 0.9 or higher, and each item and the entire survey were evaluated as excellent ^(30)^. The translated Japanese HCAHPS is available for free at https://mhlw-grants.niph.go.jp/system/files/report_pdf/202022021B-sogo.pdf.

### Association between patient hospital rating and HCAHPS items

#### Study design

We conducted a cross-sectional study of survey results obtained from January 2020 to September 2021, from patients who experienced hospitalization in a recent year. Samples were extracted from survey results and provided by a commercial survey company (Japan Management Association, Tokyo, Japan). The AHRQ has published guidelines for the selection and exclusion criteria, as follows. Selection criteria: (1) age 20 years or older; (2) spent at least one night in the hospital (admission after midnight); and (3) the questionnaire was distributed to patients within 48 hours to 6 weeks (42 days) of discharge. Exclusion criteria: (1) a primary diagnosis of psychiatric MS-DRG at discharge (patients whose primary diagnosis is a medical, surgical, or obstetric condition and who do not have a complex psychiatric diagnosis must be excluded); (2) patients who do not permit disclosure of their information; (3) court/legal patients (prisoners); (4) patients whose current residence is abroad; (5) patients discharged to hospice; (6) patients discharged to nursing homes or long-term care facilities; and (7) patients with cognitive decline. In this study, the same inclusion and exclusion criteria were adopted when collecting patient data. The sample size of 300 was calculated with reference to Hair (2009) ^(31)^. As Hair (2009) indicates that the ratio of the number of subjects to the number of questionnaire items is 10:1, the minimum number of subjects needed to clarify the construct validity of the questionnaire with 26 questions was assumed to be 260. Assuming that 10% of the respondents did not reply, the number of respondents was set to 300. Additionally, patients were selected from multiple institutions all over Japan. Consequently, we collected patients from 41 of 47 prefectures, with population demographics similar to the whole population of Japan.

#### Data analyses

The association between the patient rating of the hospital (“highly rated” or “poorly rated”) and other items obtained from the questionnaire was measured. To measure their association, we first dichotomized the patient hospital rating scale (Q18: what number would you use to rate this hospital during your stay?) into 0-7 as “poorly rated,” or 0, and 8-10 as “highly rated,” or 1. We then measured the association between this hospital rating with the 30 items from the questionnaire: nurse’s communication (Q1-Q4), doctor’s communication (Q5-Q7), hospital environment (Q8, Q9), patient’s experience during the hospital stay (Q10-Q14), patient’s experience at the time of discharge (Q15-Q17), patient’s experience after discharge (Q20-Q22), patient’s information (Q23-Q31), and overall evaluation (Q19). Questionnaire items with multiple-choice answers were dichotomized into 0 (no) and 1 (yes) for analyses. For the questionnaire items asking about patients’ hospitalization reasons (main disease), patients’ responses were dichotomized into 0 (nonmalignant) and 1 (malignant condition).

Statistical analyses of the associations were carried out in two steps: first, we used the Pearson’s chi-squared test of association to test the relationship between patients’ hospital ratings and other variables; and second, we used multivariate logistic regression to generate an adjusted odds ratio (AOR) with a 95% confidence interval (CI). The statistical significance was set at p < 0.05. The logistic models’ goodness of fit and discrimination ability were confirmed using the Hosmer-Lemeshow test. Data were analyzed using Stata 17 (StataCorp LLC, College Station, TX, USA). Ethical approval was obtained from the Research Ethics Committee of St. Luke’s International Hospital, Tokyo, Japan, on December 19, 2019 (Research number: 19-R157).

Given the nature of our study, we could not rule out the risk of recall bias. To address this potential bias, we limited the recall period to a maximum of 6 weeks from questionnaire distribution to ensure that the patients could recall their recent experience.

## Results

Of the 300 respondents, 182 (60.7%) were male and 118 (39.3%) were female. The mean age of the respondents was 61 years (standard deviation (SD): 16). The frequency of patients’ high and poor hospital ratings were 207 (69%) and 93 (31%), respectively. Of the 37 variables, 5 variables (Q11, 13, 14, 16, 17) had missing data. However, no missing data were identified for the outcome variable and key variables (n = 300).

A high patient hospital rating is significantly associated with satisfaction in doctor communication, especially for doctors giving an understandable explanation to patients (highly rated and poorly rated hospitals: 204 (98.6%) and 73 (78.5%), respectively; p < 0.001) and satisfaction in patients’ understanding of their responsibilities after discharge (highly rated and poorly rated hospitals: 204 (98.6%) and 82 (88.2%) respectively; p < 0.001) (Table 1).

**Table 1.** Characteristic Differences of Patients’ Hospital Ratings among Hospitalized Patients.

Variable			Patients’ hospital ratings			
			Total	Poor	High	p
Sex	Male		182 (60.7)	57 (61.3)	125 (60.4)	0.882
	Female		118 (39.3)	36 (38.7)	82 (39.6)	
Age (years)			61.20 (±16.257)	57.98 (±17.043)	62.65 (±15.718)	0.011
Nurse’s communication	Given courtesy and respect		277 (92.3)	76 (81.7)	201 (97.1)	<0.001
	Listened well		277 (92.3)	75 (80.6)	202 (97.6)	<0.001
	Explained well		277 (92.3)	77 (82.8)	200 (96.6)	<0.001
	Received help when needed		203 (67.7)	62 (86.1)	141 (94)	0.049
Doctor’s communication	Given courtesy and respect		278 (92.7)	73 (78.5)	205 (99.0)	<0.001
	Listened well		271 (90.3)	68 (73.1)	203 (98.1)	<0.001
	Explained well		277 (92.3)	73 (78.5)	204 (98.6)	<0.001
Hospital environment	Room/bathroom hygiene was maintained		284 (94.7)	84 (90.3)	200 (96.6)	0.025
	Peaceful at night		259 (86.3)	72 (77.4)	187 (90.3)	0.003
Experience during hospital stay	Required help when using the bathroom		56 (18.7)	22 (23.7)	34 (16.4)	0.137
	Got help as soon as it was required		244 (81.3)	20 (90.9)	32 (94.1)	0.649
	Started new medication		171 (57.0)	53 (57.0)	118 (57.0)	0.998
	Understandable explanation on new medication		171 (57.0)	43 (81.1)	114 (96.6)	<0.001
	Understandable explanation on new medication side effects		171 (57.0)	36 (67.9)	93 (78.8)	0.126
Experience at discharge	Discharge destination	Own home	289 (96.3)	88 (94.6)	201 (97.1)	0.176
		Someone else’s home	2 (0.7)	0 (0)	2 (1.0)	
		Others	9 (3.0)	5 (5.4)	4 (1.9)	
	Discussed help after discharge		204 (68.0)	41 (46.6)	163 (80.3)	<0.001
	Received information about symptoms in written form		222 (74.0)	52 (59.1)	170 (83.7)	<0.001
Overall evaluation	Would recommend this hospital		267 (89.0)	64 (68.8)	203 (98.1)	<0.001
Patient’s experience after discharge	Preference for needs after discharge was considered		275 (91.7)	75 (80.6)	200 (96.6)	<0.001
	Understood responsibilities after discharge		286 (95.3)	82 (88.2)	204 (98.6)	<0.001
	Understood the use of medication (s)		237 (79.0)	73 (92.4)	164 (97)	0.098
Patient’s information	Admitted through ER		66 (22.0)	22 (23.7)	44 (21.3)	0.643
	Overall health	Excellent	19 (6.3)	5 (5.4)	14 (6.8)	0.356
		Very good	42 (14.0)	10 (10.8)	32 (15.5)	
		Good association	87 (29.0)	26 (28.0)	61 (29.5)	
		Fair	124 (41.3)	39 (41.9)	85 (41.1)	
		Poor	28 (9.3)	13 (14.0)	15 (7.2)	
	Overall mental health	Excellent	24 (8.0)	4 (16.7)	20 (83.3)	0.154
		Very good	70 (23.3)	17 (18.3)	53 (25.6)	
		Good	90 (30.0)	33 (35.5)	57 (27.5)	
		Fair	98 (32.7)	31 (33.3)	67 (32.4)	
		Poor	18 (6.0)	8 (8.6)	10 (4.8)	
	Level of schooling	Up to compulsory education	16 (5.3)	8 (8.6)	8 (3.9)	0.07
		High school graduate/equivalent	91 (30.3)	21 (22.6)	70 (33.8)	
		2-year associate degree	28 (9.3)	11 (11.8)	17 (8.2)	
		Diploma degree	30 (10.0)	14 (15.1)	16 (7.7)	
		4-year college degree	126 (42.0)	37 (39.8)	89 (43.0)	
		More than 4-year college degree	9 (3.0)	2 (2.2)	7 (77.8)	
	Admitted to hospital for malignancy/nonmalignancy		41 (13.7)	8 (8.6)	33 (15.9)	0.087
	Had surgery during hospital stay		179 (59.7)	52 (55.9)	127 (61.4)	0.374
	Admitted to a university hospital		121 (40.3)	14 (15.1)	45 (21.7)	0.178

Table 2 illustrates the logistic results of the association between patient rating and 14 items from the questionnaire (nurse communication, doctor communication, hospital environment, experience during the hospital stay, discharge information, after discharge) and patient’s age. A high patient hospital rating was significantly associated with the patient’s age (AOR: 1.02; 95% CI: 1.00-1.04; p = 0.02), doctors listening to patients carefully (AOR: 10.47; 95% CI: 3.17-34.58; p < 0.001), and the patient’s satisfaction with the discussion regarding the responsibilities after discharge (AOR: 3.53; 95% CI: 1.96-6.36; p < 0.001). The nurse’s communication showed an odds ratio (OR) in the direction indicating an association (AOR: 2.43; 95% CI: 0.72-8.24, p = 0.155), but there was no clear association (Table 2).

**Table 2.** Factors Associated with Hospital Ratings among Hospitalized Patients.

		95% CI				95% CI			
	Variable	Crude OR	Lower	Upper	p	Adjusted OR	Lower	Upper	p
	Age	1.02	1.00	1.03	0.022	1.02	1.00	1.04	0.019
Nurse’s communication	Given courtesy and respect	7.49	2.85	19.75	<0.001
	Listened well	9.70	3.48	27.04	<0.001	2.43	0.72	8.24	0.155
	Explained well	5.94	2.35	14.99	<0.001
	Received help when needed	2.53	0.98	6.53	0.055
Doctor’s communication	Courtesy and respect	28.08	6.41	123.10	<0.001
	Listened well	18.66	6.27	55.53	<0.001	10.47	3.17	34.58	<0.001
	Explained well	18.63	5.38	64.55	<0.001
Hospital environment	Room/bathroom hygiene were maintained	3.06	1.10	8.49	0.032
	Peaceful at night	2.73	1.40	5.33	0.003
Experience during stay	Understandable explanation on new medication	6.63	1.97	22.26	0.002
Discharge information	Discussed help after discharge	4.67	2.71	8.04	<0.001	3.53	1.96	6.36	<0.001
	Received information about symptoms in written form	3.57	2.03	6.28	<0.001
After discharge	Preference for needs after discharge was considered	6.86	2.75	17.08	<0.001
	Understood responsibilities after discharge	9.12	2.48	33.54	<0.001

## Discussion

### Summary of main results

The results of the association analyses between the patient’s rating of the hospital and the HCAHPS^Ⓡ^ items suggested that a high hospital rating was significantly associated with the patient’s satisfaction with care provided at the hospital, particularly with doctors’ communication (AOR: 10.47; p < 0.001) and patients being able to understand their responsibilities after discharge (p < 0.001). The patient’s age was another factor associated with the hospital rating. From the association results, we can conclude that the patient’s age, doctor’s communication, and patient’s knowledge on their roles after discharge are associated with the patient’s satisfaction, measured using the patient rating of the hospital. As for the nurse’s communication, although no clear significant association was observed (p = 0.155), the direction of the OR was large (AOR: 2.43), which indicates an association and suggests that we cannot rule out some potential for the association between the nurse’s communication and the patient’s satisfaction.

Regarding the association analysis, our results were consistent with those from previous literature; specifically, high-quality communication, coordination, and retention of information had a positive association with patients’ satisfaction ^(32), (33)^. Furthermore, the significant association highlights the importance of efficient doctor communication for high patient satisfaction. As for discharge planning, previous literature reviews have pointed out the effectiveness in reducing the length of stay and readmission to hospital ^(34)^, and improvements in quality of life ^(35), (36)^ and satisfaction for both staff ^(37)^ and patients ^(36), (37), (38), (39)^. Our study findings were consistent with these findings, as we found a significant association between patients’ hospital ratings and patients’ satisfaction when they discussed having help after discharge with the medical staff during their hospital stay. Regarding age, a study concluded that the patient’s age affects their satisfaction, in association with the change in communication methods with doctors by age groups ^(40)^. Our findings were consistent with those from previous literature in that the older the patients were, the better they tended to rate the hospital; however, the possibility of other underlying factors that may affect the age and patient satisfaction association, such as communication methods, cannot be ruled out ^(27)^. Moreover, the age-patient rating relationship is rather controversial, with some literature demonstrating that patient rating after the age of 65 years has a decreasing trend ^(41)^. Therefore, the undelaying factors that may affect the age-patient rating relationship is needed for a better understanding of this item.

### Limitations

There are a few limitations to our study. First, as we recruited panel members to recall their experience at the hospital within a year, we cannot rule out the possibility of a recall bias. However, we have attempted to mitigate this bias by restricting the date of response of the questionnaire to 6 weeks post hospitalization, as stated in the selection criteria at data collection. The second limitation is the inability to stratify patients by different reasons for hospitalization and by hospital function. As for the hospitalization reason, we could dichotomize the results into only malignant or nonmalignant conditions. If we could have retrieved more accurate data on the reason for patients’ hospitalization, we may have been able to see an association by stratified groups. Apart from the hospitalization reason, we could not stratify the data into different hospital functions, such as acute hospitals, teaching hospitals, chronic hospitals, and so on. Our strategy to mitigate this limitation was to exclude the recruitment of patients hospitalized in nursing homes and care facilities, to collect data for acute hospitals. However, we believe that the limitation still exists as we could not get further data on the hospital functions. The CMS Hospital Performance Reports of the USA has stratified data publicly available (CMS, 2020). Therefore, by stratifying by hospital function, we may have also obtained some association results. Further research needs to collect these two groups of data to identify the factors associated with patients’ satisfaction. Although we had some missing data, no missing data were identified for the outcome measure and key variables. Additionally, in our multivariate logistic regression analyses, missing data were less than 5%. Hence, we evaluated that missing data were handled well in our study and believe that we minimized the effect that missing data may have had on our study results. Lastly, we cannot rule out the possibility of potential confounders, such as patient severity, comorbidities, and etiology. As we could not retrieve any data, we expect further studies to address this issue.

### Future implications

A study conducted among Japanese doctors revealed that the doctor-patient relationship, in the context of communication, has challenges, especially regarding the unequal positions of patients and doctors, where patients become less dominant in the relationship ^(42)^. This can cause inequality and inability to maintain a positive relationship, and for patients to feel as though they cannot communicate with their doctor when in need, leading to a decrease in patient satisfaction. In the global context, although some differences in communication styles and context were apparent for doctors in the USA and Japan, there were similar communication methods for doctors, such as the length of time spent with patients and physician versus patient ratios of questions ^(42)^. With the realization of the importance of doctor communication to patient satisfaction, a strong emphasis should be placed on improving doctor-patient communication globally.

We also found that adequate discharge planning is important in improving patient satisfaction. On this note, Japanese patients are strongly influenced by cultural superstitions, with more patients wishing to be discharged on what is considered a lucky day (Taian), instead of an unlucky day (Butsumetsu) ^(43)^. Considering these cultural factors, in addition to sociodemographic factors, long-term care insurance, medication, and welfare services, is also of great importance in improving patient satisfaction in the Japanese setting ^(43)^.

Furthermore, our findings suggest that the patient’s age, doctor’s communication, and patient’s knowledge on their roles after discharge, as well as the nurse’s communication, are key factors that contribute to the patient’s ratings of the hospital. This finding allows us to further understand the roles and responsibilities of medical staff on the patient’s hospital rating and satisfaction outcomes. An emphasis on improving the quality of care, focusing on these specific factors, can increase the patients’ ratings of the hospital.

As the HCAHPS questionnaire has been translated into various languages and used worldwide, the development of a Japanese translation of the HCAHPS will enable comparisons of patient satisfaction ratings in Japan and overseas. In addition, the HCAHPS can provide material for proposing patient-centered healthcare policies by surveying patient satisfaction using a validated questionnaire. Moreover, this study is generalizable for the Japanese population, as we collected data from multiple institutions all over Japan (41 of 47 prefectures), with similar population demographics. Thus, we believe that this is a national representative study of the Japanese population, increasing the external validity of our study results.

### Conclusions

The association analysis demonstrated a significant improvement in patients’ ratings of the hospital, in association with doctor communication and discharge planning. Further research is needed to determine the factors that most likely contribute to patients’ ratings of the hospital.

## Article Information

### Conflicts of Interest

None

### Sources of Funding

This work was supported by the Health Labor Sciences Research Grant “Nationwide survey research for development of healthcare quality assessment” (Principal Investigator: Dr. Fukui Tsuguya), grant Number 19IA2013. The grant agency was not involved in data collection; analysis or interpretation; trial design; resident recruitment; or any aspect pertinent to the study.

### Acknowledgement

We would like to express our gratitude to our participants for sharing their opinions on the assessment of the translated HCAHPS^Ⓡ^.

### Author Contributions

H.N., N.Y., S.O., and O.T. conceptualized and designed the study. H.N. and N.Y. analyzed the data. H.N. wrote the original manuscript. S.O. reviewed and edited the manuscript. All authors contributed to drafting and revising the intellectual content. All authors read and approved the final manuscript.

### Ethics Approval and Consent to Participate

Ethics approval for this survey was granted by St. Luke’s International University (19-R157). We carried out this survey in accordance with the Ethical Guidelines for Medical and Health Research Involving Human Subjects. Researchers used the informed consent form to explain the research objectives and methods and the principles of voluntary participation, data management, and privacy protection to those who met the inclusion criteria. We surveyed the participants who consented to participate in this research. Informed consent was obtained from all participants. The study process was carried out in accordance with the Declaration of Helsinki.
